# Diverse Wheat-Alien Introgression Lines as a Basis for Durable Resistance and Quality Characteristics in Bread Wheat

**DOI:** 10.3389/fpls.2020.01067

**Published:** 2020-07-17

**Authors:** Eva Johansson, Tina Henriksson, Maria Luisa Prieto-Linde, Staffan Andersson, Rimsha Ashraf, Mahbubjon Rahmatov

**Affiliations:** ^1^ Department of Plant Breeding, The Swedish University of Agricultural Sciences, Alnarp, Sweden; ^2^ Lantmännen Lantbruk, Svalöv, Sweden

**Keywords:** agronomic performance, baking quality, breeding, disease and pest resistance, *Leymus* spp., *Secale cereale* L., *Triticum aestivum* L.

## Abstract

Wheat productivity has been significantly improved worldwide through the incorporation of novel genes from various gene pools, not least from wild relatives of wheat, into the commonly cultivated bread and durum wheat. Here, we present and summarize results obtained from a diverse set of wheat-alien introgression lines with mainly introgressions of rye, but also of *Leymus* spp. and *Thinopyrum junceiforme* into bread-wheat (*Triticum aestivum* L.). From this material, lines carrying 2RL were found with good agronomic performance and multiple resistance not least towards several races of powdery mildew. A novel resistance gene, one of few showing resistance towards all today identified stem rust races, designated *Sr59*, was also found originating from 2RL. Lines with multiple introgressions from 4R, 5R, and 6R were found resistant towards the majority of the stripe rust races known today. Due to lack of agricultural adaptation in these lines, transfer of useful genes into more adapted wheat material is a necessity, work which is also in progress through crosses with the CS*ph1b* mutant, to be able to only transfer small chromosome segments that carry the target gene. Furthermore, resistance towards Russian wheat aphid was found in lines having a substitution of 1R (1D) and translocations of 3DL.3RS and 5AL.5RS. The rye chromosomes 1R, 2R, and 6R were found responsible for resistance towards the Syrian Hessian fly. High levels of especially zinc was found in several lines obtained from crosses with *Leymus racemosus* and *Leymus mollis*, while also some lines with 1R, 2R, or 5R showed increased levels of minerals and in particular of iron and zinc. Moreover, lines with 1R, 2R, 3R, and *Leymus* spp. introgressions were also found to have a combination of high iron and zinc and low cadmium concentrations. High variation was found both in grain protein concentration and gluten strength, measured as %UPP, within the lines, indicating large variation in bread-making quality. Thus, our study emphasizes the impact that wheat-alien introgression lines can contribute to current wheat lines and shows large opportunities both to improve production, resistance, and quality. To obtain such improvements, novel plant breeding tools, as discussed in this paper, opens unique opportunities, to transfer suitable genes into the modern and adapted wheat cultivars.

## Introduction

Wheat is one of the three major crops of importance for food security worldwide, the other two being rice and maize (FAO, 2016). Bread wheat (*Triticum aestivum* L.) is a hexaploid and the most commonly cultivated species of wheat (95%), belonging to the tribe Triticeae and the family Poaceae (McFadden and Sears, 1946; Dubcovsky and Dvorak, 2007). The second most commonly cultivated form of wheat is durum wheat (*Triticum durum* L.), contributing 5% to the total production (Dubcovsky and Dvorak, 2007). In total, wheat contributes 20% of the total calories and proteins consumed by the human population, thereby contributing to a higher total protein intake than the whole total meat consumption summed (Shewry and Hey, 2015).

Due to the high contribution of wheat to the daily human food intake, human food security is highly vulnerable to the increasing threats to wheat production from climate change, including global warming (Steenwerth et al., 2014). Wheat yield is also negatively affected by abiotic and biotic stresses resulting in economic losses to farmers (Husenov et al., 2020). The population growth predicted to be more than 9 billion people worldwide in 2050, result in additional demand on food production, simultaneously bringing an increasing competition for arable land for food production (FAO, 2016). To meet these challenges, novel wheat cultivars are urgently needed, adapted to contribute high yield under sustainable and demanding cultivation conditions (Shiferaw et al., 2013). For this purpose, novel plant breeding methodologies have to be developed in order to most beneficially use available genetic resources and smart and rapid plant development to produce the needed wheat materials in time to cope with needs and challenges.

Plant breeding to obtain sustainable, high resistance and high-quality crops are dependent on suitable genes for the wanted traits. For many traits, such genes are available within the breeding material in on-going breeding programs for the crop and will be easily transferred by breeders through ordinary crossing schemes. However, domestication and breeding practices have reduced the presence of rare and favorable allelic variation to biotic and abiotic stresses and environmental changes originally found in the wild relatives (Tanksley and McCouch, 1997; Singh et al., 2018). Therefore, wild relatives, landraces, and close relatives of wheat are a unique source of novel genetic variations for introgression into modern cultivars (Molnár-Láng et al., 2015). For wheat, several useful transfers of genes from landraces have been reported including e.g. the *Rht* dwarfing genes, the powdery mildew resistance gene *Pm24*, and several biotic and abiotic stress resistance genes (Kihara, 1983; Zeven, 1998; Huang and Röder, 2011; Cavanagh et al., 2013; Singh et al., 2018). Also, genes have been transferred to wheat from non-*Triticum* (alien) species, where transfers from e.g. rye (*Secale cereale*) have resulted in widely cultivated wheat cultivars (McIntosh et al., 1995; Friebe et al., 1996). The most successful alien transfer into the wheat genome is that of the 1RS chromosome segment, in the form of 1AL.1RS, 1BL.1RS, and 1DL.1RS translocations (Rabinovich, 1998; Mago et al., 2015), contributing several resistance genes for powdery mildew, leaf, stripe and stem rusts. Out of them, the 1BL.1RS wheat-rye translocation has contributed immensely to global wheat production as a source of resistance genes (*Sr31*/*Yr9*/*Lr26*/*Pm9*) to wheat fungal diseases (Schlegel, 2020), but it is also known to contribute weak and sticky dough (Dhaliwal et al., 1988). Rye is a unique source of many important traits for wheat improvement, e.g. the resistance genes *Sr27*, *Sr50*, *Sr1RS* ^Amigo^, *Lr25*, *Lr45*, *Pm7*, etc. have been identified from rye (The et al., 1991; Marais and Marais, 1994; McIntosh et al., 1995; Friebe et al., 1996), although these genes have contributed limitedly to agricultural production until now. More recently, some novel resistance genes from rye i.e. *Sr59*, *Yr83*, and *Pm56* have been introgressed into wheat (Rahmatov et al., 2016a; Hao et al., 2018; Li et al., 2020), which may be used as durable sources against fungal diseases. Herbicide-resistant evolution is challenging weed management; therefore, the allelopathic potential is a good solution to mitigate weed management in crop production. Bertholdsson et al. (2012), reported that rye is an excellent source of allelopathic potential that can be used for wheat breeding. In addition, Iron (Fe) and Zinc (Zn) deficiency are severely affecting human health, causing several physiological disorders, symptomatic anemia, stunting, etc., and therefore high content in staple crops such as wheat are of outmost importance (Johansson et al., 2014; Johansson et al., 2020). The recent great advancements in genomic and cytogenetic tools open opportunities to transfer alien resistance genes to wheat, simultaneously avoiding linkage drag issues.

The present paper is focusing on opportunities and challenges of the use of a diverse set of wheat-alien introgression lines with mainly introgressions of rye, but also of *Leymus* spp. and *Thinopyrum junceiforme* into bread-wheat (*T. aestivum* L.). This provides useful insight into the identification and characterization of wheat-alien introgression lines based on several studies through diseases and pests screening, agronomic performances and molecular markers. Resistances and quality characteristics of wheat within this material, connections to introgressed chromosomes, localization of genes, and status for transfer of these genes are described here. Finally, a short overview is given as to the impact of novel breeding strategies for the use of alien germplasm in modern breeding.

## Materials and Methods

### Plant Materials

A set of winter and spring wheat-alien introgression lines maintained at the Plant Breeding Department at the Swedish University of Agricultural Sciences were used in different part of the hereby presented studies. These lines were developed by crossing and backcrossing strategies during 1980 to 2000 by the late Professor Arnulf Merker at the Swedish University of Agricultural Sciences (**Table 1**). The wheat-alien introgression lines used for the present paper contained rye chromosomes with 1R, 2R, 3R, 4R, 5R and 6R in the form of a single disomic substitution wheat–rye translocations such as 1DL.1RS, 1BL.1RS, 2BS.2RL, 3DL.3RS and 5AL.5RS, lines with multiple combinations of rye chromosome substitutions such as 1R + 2R, 1R + 3R, 1R + 6R, 5R + 4R + 7R and 1R + 6R + 4R + 7R (Merker, 1979; Merker and Rogalska, 1984), and lines with introgressed chromatin from *Leymus mollis*, *Leymus racemosus*, and *T. junceiforme* (Ellneskog-Staam and Merker, 2001; Ellneskog-Staam and Merker, 2002). The full material used has previously been completely described in Rahmatov (2016) and Rahmatov et al. (2017).

**Table 1:** Wheat-alien introgression lines and respective parents evaluated in this study.

Cross/Pedigree	Plant habit	No. of lines	Type	Reference
Triticale^a^	Spring and winter	5	*×Triticosecale*	Forsstrom and Merker, 2001
Wheat^a^	Spring and winter	8	*Triticum aestivum* and *Tr. carthlicum*	Forsstrom et al., 2002
Sv 876012 x H	Winter	37	Wheat–rye introgressions	Forsstrom and Merker, 2001
Sv 876032 x H x K	Winter	54	Wheat–rye introgressions	Forsstrom and Merker, 2001
Sv 856003 x H	Winter	6	Wheat–rye introgressions	Forsstrom and Merker, 2001
Sub 1R + 2R	Winter	42	Wheat–rye introgressions	Forsstrom and Merker, 2001
Malysh	Winter	6	Wheat–rye introgressions	Merker, 1984
Starke × Otello	Winter	7	Wheat–rye introgressions	Merker, 1984
Uno × Holme	Winter	8	Wheat–rye introgressions	Merker, 1984
Triticale VT828041	Spring	6	Wheat–rye introgressions	Merker, 1984
Triticale Drira	Spring	23	Wheat–rye introgressions	Merker, 1984
Triticale Beagle	Spring	12	Wheat–rye introgressions	Merker, 1984
Triticale VT83 615	Spring	2	Wheat–rye introgressions	Merker, 1984
Triticale VT83 591	Spring	4	Wheat–rye introgressions	Merker, 1984
Triticale VT 82 8039	Spring	5	Wheat–rye introgressions	Merker, 1984
3R BB14 (Cimmyt 1974)	Spring	4	Wheat–rye introgressions	Merker, 1984
*Leymus mollis*	Winter	42	Wheat–*L. mollis*introgressions	Merker and Lantai, 1997
*Leymus racemosus*	Spring	22	Wheat–*L. racemosus* introgressions	Merker and Lantai, 1997
*Th. junceiforme*	Spring	16	Wheat–*T. junceiforme* introgressions	Merker and Lantai, 1997
^a^Parental cultivars and Lines	309	TOTAL	

### Field Trials

A total of 180 of the winter wheat lines and 57 of the spring wheat lines were evaluated by field trials for multiple resistance and agronomic performance during two executive seasons, 2014 and 2015, in Svalöv, Sweden and in Harzhof and Laberweinting in Germany. During these seasons, the lines were continuously evaluated and scored (scale 1–9) for lodging (winter wheat) and presences of diseases (spring and winter wheat). Comparisons of presence of diseases and alien material were carried out (Andersson et al., 2016).

### Diseases Screening

Stem rust seedling resistance assays with ten *Pgt* races and adult plant responses with three *Pgt* races (TTKSK + TTKST, TKTTF and MCCFC), were carried out on 185 and 94 of the winter and spring wheat-alien introgression lines under field conditions following the procedure described in Hysing et al. (2007) and Rahmatov et al. (2015); Rahmatov et al. (2016a, b). For the stripe rust evaluations, 189 of the winter and 73 of the spring wheat-alien introgression lines were tested in the seedling and adult plant stages. Twelve stripe rust races with different virulence/avirulence combinations and geographic origins were used for screening at the seedling stage along with adult plant evaluations in the field according to Rahmatov et al. (2017). Hysing et al. (2007), evaluated a set of 2BS.2RL wheat-rye translocation lines against stripe rust, leaf rust, and powdery mildew races.

### Hessian Fly and Russian Wheat Aphid Screenings

A total of 57 spring and 185 winter wheat-alien introgression lines were evaluated in 2011 and 2012 at the seedling stage against Hessian fly (HF) and the Russian wheat aphid (RWA) in collaborations with ICARDA. In brief, the rearing rooms for HF experiments were kept at 20°C, Rh 70–80%, and the cycle of 16/8 h light/dark was used. Six or ten seeds per wheat accession were sown in hill plots in metal flats 55 × 45 × 10 cm, in total 48 accessions per box plus controls, in a mixture of soil:sand:peat (2:1:1). After 5–6 days, at the one-leaf stage, infestation by HF was done with about 30 females and 10 males under net for 3–4 days (El Bouhssini et al., 2013). The scoring took place 20 days after infestation, with the number of resistant and susceptible plants per accession. The first screening was conducted in the spring and winter materials in 2011, and a second screening was only conducted in the winter materials in 2012. Based on these two screenings, lines with 100% resistance reaction to HF were selected for further confirmations in four separate screenings.

The RWA biotype was collected from Tel Hadya, Syria, and thereafter reared on the susceptible wheat cultivar (Andersson et al., 2015). The experiments were carried out in a greenhouse at 19–20°C, with light/dark photoperiod 16/8 h and relative humidity of about 60%. The accessions were planted in a randomized (alpha design) order together with susceptible and resistant controls in each planting tray, in a mixture of soil, sand, and peat (2:1:1). An evaluation was done when symptoms were seen on susceptible checks, using the ICARDA RWA damage scale with a 1–3 scale for leaf rolling (LR) and 1–6 scale for leaf chlorosis (LC) (El Bouhssini et al., 2011). In the second advanced screening, selected accessions from the first screening results were repeated at four separate times (Andersson et al., 2015).

### Allelopathic Potential of Wheat-Alien Introgression Lines

Allelopathic potential of the wheat–rye introgression lines were tested according to Bertholdsson et al. (2012). In this study, seeds of *Chenopodium alba*, *Lolium perenne*, *Brassica napus*, *Lactuca sativa*, *Eruca sativa*, *Sinapis indicum* and *Sinapis alba* were used to find high root growth inhibition when grown together with rye. In this investigation, four pregerminated cereal seedlings were planted along the wall of 400-ml Phytotech tissue culture vials (bottom diameter 75 mm) filled with 20 ml 0.35% water agar, and eight pregerminated mustard seedlings (*S. alba* cv. Medicus) were planted in a circle in the center of the vials. The experiment was tested in four replicates, and the dry weight of the shoot and root were measured (Bertholdsson et al., 2012).

### Analysis of Grain Samples for Micronutrients Concentration and Protein Composition

A total of 40 of the lines were evaluated for micronutrients (e.g. Iron, Zinc, and Cadmium) content with Inductively Coupled Plasma Mass Spectrometry (ICPMS) at the University of Minnesota, similarly as described in Hussain et al. (2010) and Moreira-Ascarrunz et al. (2016). Briefly, all samples were ashed in a muffle furnace for 12 h at 485°C. Then, the ash was dissolved in 5 ml of 20% HCl followed by dilution with 5 ml of deionized water. The ICPMS provides concentration assays for several microelements, including zinc, iron, and cadmium in mg/Kg.

The complete set of winter wheat alien translocation lines were analysed with SE-HPLC according to Johansson et al. (2001) to evaluate the total amount of SDS-extractable proteins (TOTE) and percentage of unextractable polymeric protein in total polymeric protein (%UPP). A high correlation is known to exist between TOTE and grain protein concentration and between %UPP and gluten strength (Malik et al., 2011; Malik et al., 2013) and thereby this methodology can be used to understand relationships with bread-making quality (Hussain et al., 2012; Hussain et al., 2013; Vazquez et al., 2019).

### Statistical Analyses

The statistical software SAS 9.3 (SAS, 2011) was used for principal component analyses (PCA) calculations to understand relationships between minerals and protein factors with evaluated wheat-alien introgression lines. In order to understand and visualize the distribution and relationship between variables and factors evaluated, principal component analysis (PCA) can be applied to orthogonally represent the variables in a data matrix vector. PCA is known to show the distribution of dependent variables and independent factors, in a loading and score plot, respectively (Wold et al., 1987). Values of content of Iron, Zinc and Cadmium were calculated by mini tab for wheat, Triticale, wheat–rye and wheat-*Leymus* lines and presented as boxplots with lowest and highest observations as well as lower and upper quartile and median.

## Results

### Multiple Resistance and Agronomic Performance

**Figure 1:**
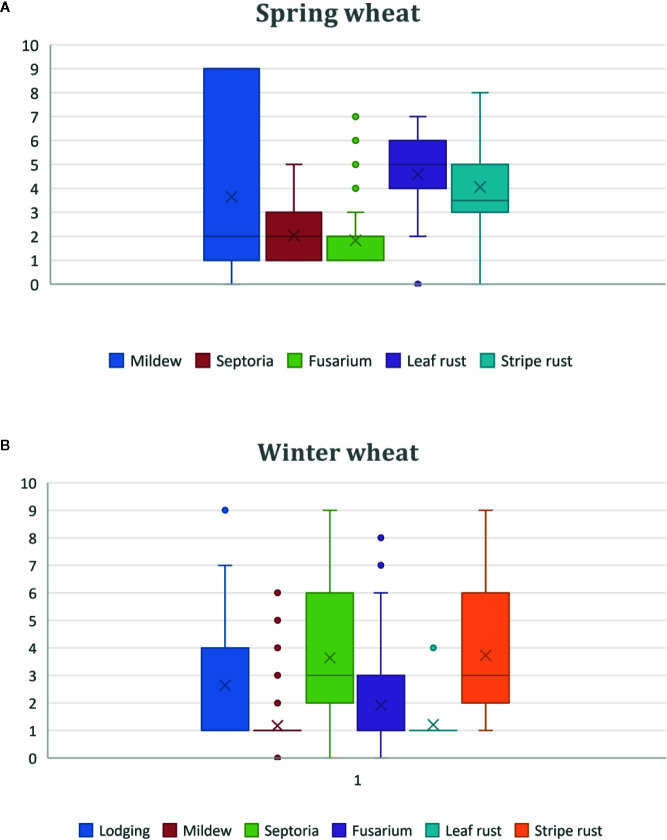
Boxplots showing variation in lodging and various diseases based on scoring of the material from 0 to 9, in wheat alien introgression lines of **(A)** Spring wheat, and **(B)** Winter wheat, from field trials during two years in Sweden and Germany. In each boxplot, five bars are represented, indicating smallest observation, lower quartile, median, upper quartile, and largest observation, respectively. X marks the mean value.

The lines showed varying agronomic performance, with some lines being almost comparable to currently grown wheat in Sweden while others differed largely. Large variation was found in the material both for lodging and presence of diseases (**Figure 1**). However, the majority of the winter wheat lines had strong stem and with limited lodging, thus making them of interest as a source of lodging resistance (**Figure 1B**). Presence of 1R, 2R, 3R, 5R, 1R + 6R and *L. racemosus* correlated with decreased levels of infections with powdery mildew, *Zymoseptoria tritici (*causal agent of *Septoria triticae* blotch) and Fusarium head blight during field conditions. Lower levels of leaf, stem and stripe rusts infection responses were found in lines with 1R, 2R, 3R, 1R + 3R, 1R + 6R, and *L.* *racemosus*, respectively.

### Rusts Screenings

From the stem rust seedling evaluation, eleven 2R (2B), three 2R (2D), and three 3R (3D) wheat–rye disomic substitution lines, and seven wheat-*T. junceiforme* were found to carry potentially new stem rust resistant gene/s (**Table 2**). Based on the ten *Pgt* races, known resistance genes could not be postulated because their reactions did not correspond to the avirulence/virulence profile of the races tested. All lines that were resistant at the seedling stage remained resistant at the adult plant stage against races TTKSK + TTKST in Kenya and TKTTF in Turkey. Trace resistance was found in several of the lines tested at St. Paul, Minnesota, against the race MCCFC (**Table 2**), although only a few number of lines were tested due to winter type of the material and limited seed available.

**Table 2:** Stem rust seedling and adult plant resistance tests in the wheat–rye and wheat–*T. Junceiforme* introgression lines with potential sources of new stem rust resistance gene/s.

#	Chromosome	Seedling Resistance Test											Adult Plant Resistance		
		TTKSK, 1 Rep.	TTKSK, 2 Rep.	TPMKC	TTTTF	QTHJC	RKQQC	TTKST	TRTTF	TTTSK	TKTTF	MCCFC	TTKSK+ TTKST	TKTTF	MCCFC
**SLU73**	2R susbstituted 2B	2+	2	3+	;1	;01/3+	11+	2/2+	1+2/3+4	22+	1/2+	4	20MR	10MR	–
**SLU74**	2R susbstituted 2B	2+	2+	1 3+ Z	;1/1	;-1	;1	;11+	1+2/3+	22+	1/2+	4	30MR	10MR	–
**SLU75**	2R susbstituted 2B	2	2	3+	0;/0; 3+	;-1	;1	11+	33+	22+	11+	4	20MR	10MR	–
**SLU76**	2R susbstituted 2B	2	2	3+	;1	;1/2-	;11+	;11+	11+3	22+	3+	4	30MRMS	10MS	–
**SLU77**	2R susbstituted 2B	2	2	3+	2+3/4/;1	;1/1+3	;11+/2	;11+/2/3+	33+	22+	3+	4	30MRMS	10MRMS	–
**SLU78**	2R susbstituted 2B	2+	2+	3+	;/3-	1+3-	;11+3-	;11+2+	22+3+	22+	3+	4	30MRMS	10MS	–
**SLU79**	2R susbstituted 2B	2	2	3+	;/3-	;13-	;13-/3	;12+	11+/3+	22+	1	4	30MRMS	10MR	–
**SLU80**	2R susbstituted 2B	2+	2+	33+	3+	33+/;13-	33+/;13-	;11+/3+	1+2/3+	22+	3+	4	30MRMS	10MS	–
**SLU81**	2R susbstituted 2B	2+	2+	3+	3-/1;	11+	11+	1+3-	33+	22+	3+	4	40MRMS	10MS	–
**SLU82**	2R susbstituted 2B	2+	2+	3+	; 1 3-/4	;13-/3+	;13-/3+	;1/3	3+	22+	3+	4	40MRMS	10MS	–
**SLU83**	2R susbstituted 2B	2+	2+	4	; 1 3-	13-/3+	13-/3+	11+/3+	33+	22+/3+	3+	4	40MRMS	10MS	–
**SLU210**	2R susbstituted 2D	0;	0;	0;	1+	0;	;1	0;	;12	;1	1	0;	20RMR	5RMR	TR
**SLU214**	3R susbstituted 3D	0;	0;	11+	4	0;	0;	0;	;11+	3+	3+	0;	20R	10MRMS	TR
**SLU219**	3R susbstituted 3D	0;	0;	0;	0;	0;	0;	0;	0;	0;	0;	0;	5TR	5R	TR
**SLU222**	3R susbstituted 3D	0;	0;	33+	4	1+2/3-	3	0;	22+	;	33+	11+	10RMR	70S	10R
**SLU238**	2R susbstituted 2D	1	1	2	22-	12-	;1	;11+	;01	;1	1-	;01-	10R	10RMR	TR
**SLU239**	2R susbstituted 2D	1	1-	2	22-	12-	;1	;01	;01	;1	1-	;01-	20RMR	5RMR	TR
**SLU251**	Th.-Wheat	0;	0;	3+	4	33+	3+	0;1	3+	0;	3+	4	–	–	–
**SLU252**	Th.-Wheat	0;	0;	33+	3+	33+	3+	0;	3+4	0;	3+	3+	–	–	–
**SLU253**	Th.-Wheat	1	0;	33+	3+	33+	3+	1+3-	2+3-	0;	3+	3+	–	–	–
**SLU255**	Th.-Wheat	0;	0;	3+	4	33+	3+	0,1	3+	0;	3+	3+	–	–	–
**SLU256**	Th.-Wheat	0;	0;	3+	3+	33+	3+	0;	3+4	0;	3+	3+	–	–	–
**SLU274**	Th.-Wheat	0;	0;	3+	4	33+	3+	0,1	3+	0;	3+	3+	–	–	5MS
**SLU275**	Th.-Wheat	0;	0;	33+	3+	33+	3+	0;	3+4	0;	3+	3+	–	–	30MS

Seedling infection types observed based on 0–4 scale (Stakman et al., 1962). The lines with;0–2+ types considered as resistant. The lines with 3–4 types considered as susceptible. Adult plant response was evaluated based on the Cobb Scale (Peterson et al., 1948) and host response to infection based on pustule type and size (Roelfs et al., 1992). TR, Trace Resistance; R, Resistance; MR, Moderately Resistance; MRMS, Moderately Resistance to Moderately Susceptible; and MS, Moderately Susceptible. A total of 94 lines of the total material were screened for adult plant resistance, explaining the lack of data for some of the lines presented here.

The wheat-alien introgression lines showed high variability in resistance/susceptibility reactions against the twelve stripe rust isolates applied to screen for resistance genes (**Table 3**). The screening resulted in 149 lines (57% of the lines), postulated to contain a combination of known *Yr* genes e.g. *Yr1*, *Yr2*, *Yr9*, and *Yr32*. However, six of the multiple wheat–rye introgression lines with 4R, 5R and 6R were identified as highly resistant against a total of 25 stripe rust races, including the twelve used for the full material (**Table 3**). Thus, these six lines might possess a new stripe rust resistance gene/s. Molecular cytogenetic analysis showed that the 4R, 5R and 6R rye chromosomes substituted 4D, 5D and 6D wheat chromosomes. Further studies are going on for determining the underlying genetic basis of these resistance gene/s.

**Table 3:** Resistance(R)/susceptibility(S) of wheat-alien introgression lines to isolates of *Puccinia striiformis tritici*.

Isolates	6 lines fromSv 876012 × H	256 lines			
	with 4R + 5R + 6R	R	MR	MS	S
SE 205/12	R	12	23	102	119
UK 94/519	R	49	2	24	182
DK 66/02	R	84	4	29	140
Taj 01a/10	R	17	34	87	119
ER 02/03	R	174	15	54	14
DK 11/09	R	178	11	46	21
DK 71/93	R	66	34	99	56
AF 87/12	R	207	10	22	16
DK09/11	R	39	14	104	98
DK 122/09	R	6	15	126	109
SE 100/09	R	220	8	16	12
TR 34/11	R	170	41	8	32

### Aphid and Hessian Fly Resistance

**Figure 2:**
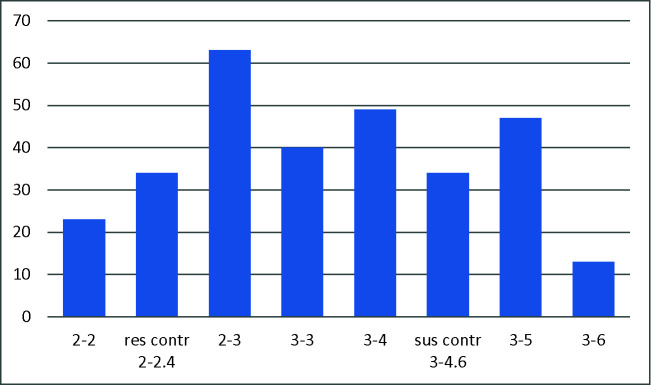
Evaluation of 235 wheat alien introgression lines for resistance against Russian wheat aphid (RWA), resulting in number of lines with different scales of resistance. Scale: leaf rolling (LR)-leaf chlorosis (LC); 1-1 and 1-2 = highly resistant, 2-2 = resistant, 2-3 = moderately resistant, 3-3 moderately susceptible, 3-4, 3-5, 3-6 = susceptible. For the susceptible and resistant controls, mean values of 34 lines are used.

Among the total of 242 evaluated lines, 235 germinated and showed a high variation in resistance to RWA (**Figure 2**). A total of 23 accessions were identified as resistant against the RWA. Resistance was found to RWA, particularly in accessions having substitutions of 1R instead of 1D [1R (1D) or 1R (1D) + 6R (6D)], in translocations to 3D or 5A (3DL.3RS and 5AL.5RS) and accessions with introgressions of *L. mollis*.

The first screening (242 lines) for HF resistance showed 11 winter and two spring wheat accessions with 100% resistance, while in the second screening, nine of the 11 winter wheat accessions were proofed with 100% resistance, which also holds true for the additional four repeated screenings (**Table 4**). These fully resistant winter wheat accessions contained 1R, 1R + 6R, 1RS + 2RL, 1RL + 2RL, 2RL, and 2R translocations or substitutions. The presence of these genes in our alien wheat material might be one explanation for the HF resistance found although the presence of full resistance in accessions with the substitution 1R.1D in winter wheat and the translocation 1RS.1DL in spring wheat indicate the presence of additional unknown resistance genes in the present material. Besides, high and partial levels of resistance with the presence of 1R, 1RS, 2R, 3R, 3RS, 4R, 5R, 6RL, and *L. racemosus* and *L. mollis* substitutions and translocations were found promising sources against HF.

**Table 4:** Accessions of Swedish winter wheat with rye substitutions and translocations showing resistance for Hessian fly at separate screenings.

Acc.No.	Subs./transl.	1st screen		2nd screen		Mean 4 screens	
		Tot pl	% inf	Tot pl	% inf	Tot pl	% inf
Kr 08-59	1R.1D	5	0	10	0	9.25	0
Kr 08-60	1R.1D + 6R.6D	5	0	10	0	8.75	0
Kr 08-76	T1RS.1BL + T2BS.2RL	2	0	10	0	9.5	0
Kr 08-79	2R.2B	5	0	10	0	9.25	0
Kr 08-89	T1RL.1BS + T2BS.2RL	5	0	10	0	9.5	0
Kr 08-90	T1RL.1BS + T1BS.2RL	2	0	10	0	9.5	0
Kr 08-91	T2RL.2BS	5	0	10	0	9	0
Kr 08-94	T2RL.2BS	5	0	10	0	10	0
Kr 08-95	T2RL.2BS	5	0	10	0	9	0
Res Cont	6RL	5	0	10	0	10	0
Sus Cont	-	5	100	10	100	10	100

### Nutritional Benefits

**Figure 3:**
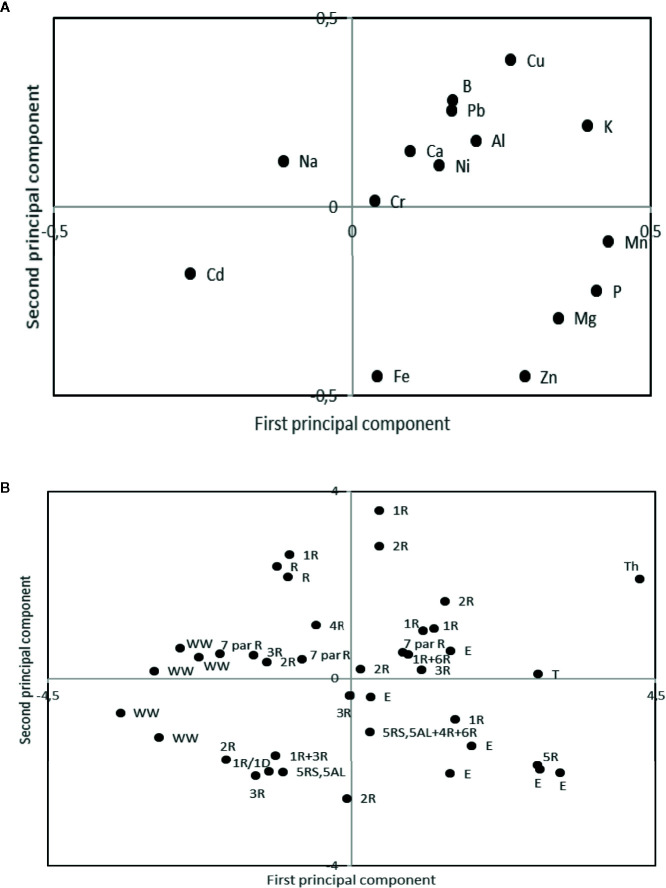
Loading **(A)** and score **(B)** plot from principal component analyses of mineral composition in winter wheat (WW), Triticale (T), Rye (R), and alien substitution and translocation lines with rye introgressions (given as R and a what type of), Thinopyrum (Th) and Leymus (E) introgressions. The first and second principal component explained 23.0 and 17.0% of the variation, respectively.

Principal component analyses indicated high levels of Cadmium (Cd) in the winter wheat lines as compared to the rest of the evaluated lines, while *Leymus* spp. was indicated as containing high levels of Iron (Fe) and Zinc (Zn; **Figure 3**). Mean values of minerals content in the different types of material (Wheat–rye introgressions, *Leymus* spp. introgressions, wheat, and triticale) verified the high content of Zn in the *Leymus* spp. introgression lines and the high Cd content in the wheat lines (**Table 5**). A relatively high Fe content was found in two of the parental wheat lines used in the present study; Sonett (57.0 mg/kg) and Prins (60.6 mg/kg). Furthermore, the triticale parents, Drira (51.8 mg/kg) and Beagle (63.3 mg/kg), were observed to contain a high level of Zn (**Table 5**). Overall, the minimum 22.7 mg/kg and maximum 64.2 mg/kg for Fe concentrations were observed in the wheat–rye introgression lines with 1R, 2R, 3R, 5R, and 6R rye chromosomes (**Table 5**). The minimum and maximum Zn concentrations produced by these wheat–rye introgression lines were 32.9 mg/kg and 89.3 mg/kg, respectively. The overall grain Cd concentration ranged from 0.02 to 0.13 mg/kg, in which the lines with low Cd concentration were observed to be 0.015 to 0.017 mg/kg in the wheat–rye introgression 1R (1D), and the lines with *L.* *mollis* and *L. racemosus* chromosomes. Interestingly, nine of the lines with a high combination of Fe (ranged from 47.4 to 64.2 mg/kg) and Zn (ranged from 53.7 to 83.4 mg/kg) concentration and low Cadmium concentration (ranged from 0.02 to 0.07 mg/kg) were detected in the wheat-rye 1R (1D), 2R (2D), 2R (2B), 3R (3B), and *L.* *mollis* and *L. racemosus* intogression lines (**Table 5**).

**Table 5:** Mean values of zinc, iron and cadmium concentrations in wheat, triticale, *Leymus* spp., wheat–rye introgression and wheat–*Leymus* spp. introgression lines.

Plant lines	Fe (mg/kg)		Zn (mg/kg)		Cd (mg/kg)	
	Mean	Range	Mean	Range	Mean	Range
Wheat (n = 5)	45.0	31.0–60.6	39.5	34.5–48.7	0.09	0.07–0.12
Rye (n = 2)	39.7	38.1–41.2	35.2	33.8–36.6	0.00	0.00–0.00
Triticale (n = 5)	37.9	29.5–45.0	48.9	38.7–63.3	0.09	0.07–0.15
*Leymus* **spp.** (n = 3)	49.4	41.4–59.1	75.8	62.4–83.4	0.02	0.00–0.02
Wheat–rye introgression (n = 22)	38.9	22.7–64.2	54.8	32.9–89.3	0.05	0.00–0.10
Wheat–*Leymus* **spp.** introgression (n = 3)	47.5	43.0–51.9	63.6	53.1–69.1	0.04	0.02–0.06

### Baking Quality

**Figure 4:**
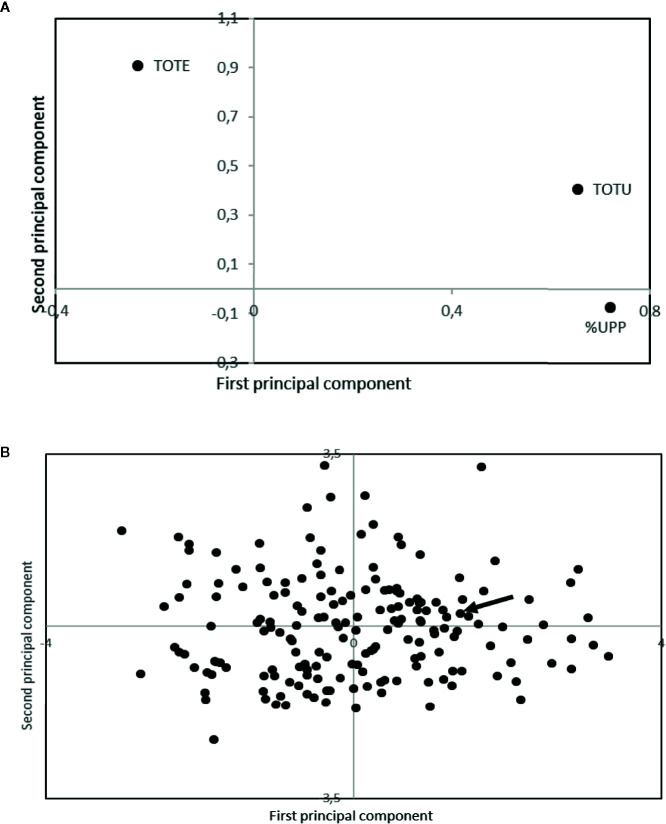
Loading **(A)** and score **(B)** plot from principal component analyses of storage protein composition from SE-HPLC. The arrow is indicating the Swedish spring wheat line, used as a standard within the analyses. TOTE, total amount of SDS-extractable proteins; TOTU, total amount of SDS-unextractable proteins; and %UPP, percentage of unextractable polymeric protein in total polymeric protein. The first and second principal component explained 58.8 and 35.6% of the variation, respectively.

The evaluated alien introgression lines showed a high level of variability both in grain protein concentration and gluten strength (**Figure 4**). A total of 40% of the lines showed a higher grain protein concentration than the standard cultivar, Dragon, while 8% of the lines showed higher gluten strength than the standard. The 10% of the evaluated lines with the highest grain protein concentration (TOTE), were all found to have either addition of chromosome 1R, 2R, 4R, and 6R or a 1R/1D translocation (**Table 6**). Several of the high grain protein concentration lines also had additions of 1R and 6R. The lines with high gluten strength (%UPP) were found either to have introgressions from *Leymus* or additions of either 1R + 2R or 1R + 4R (**Table 6**).

**Table 6:** Accessions of Swedish winter wheat with substitutions and translocations (rye = R, Leymus) showing high (in descending order) total amount of SDS-extractable protein (TOTE—correlating to grain protein concentration) and percentage of unextractable polymeric protein in total polymeric protein (%UPP—correlating with gluten strength).

TOTE			%UPP		
Acc.No.	Subs./transl.	Rel. values	Acc.No.	Subs./transl.	Values
Kr 08-10	1R, 4R, 6R, 7R	1.74	Kr 08-109	*Leymus*	86.4
Kr 08-54	1R/1D	1.70	Kr 08-107	*Leymus*	85.7
Kr 08-16	1R, 4R, 6R, 7R	1.64	Kr 08-111	*Leymus*	85.1
Kr 08-57	1R/1D	1.63	Kr 08-104	*Leymus*	84.3
Kr 08-15	1R, 4R, 6R, 7R	1.60	Kr 08-100	2RL/2BS	81.9
Kr 08-08	1R, 4R, 6R, 7R	1.60	Kr 08-28	1R + 6R	81.6
Kr 08-55	1R/1D	1.59	Kr 08-79	1R + 2R	81.0
Kr 08-09	1R, 4R, 6R, 7R	1.59	Kr 08-80	1R + 2R	80.3
Kr 08-53	1R/1D	1.54	Kr 08-110	*Leymus*	79.3
Kr 08-30	IR + 6R	1.53	Kr 08-04	1R + 4R	79.2
Kr 08-143	5R/5A	1.53	Kr 08-108	*Leymus*	78.6
Kr 08-63	1R + 6R	1.52	Kr 08-77	1R + 2R	78.4
Kr 08-75	1RS + 2RL	1.50	Kr 08-106	*Leymus*	76.6
Kr 08-76	1RS + 2RL	1.50	Kr 08-01	1R + 4R	75.3
Kr 08-82	1R + 2R	1.47	Kr 08-95	1R + 2R	75.0
Kr 08-60	IR + 6R	1.47	Dragon		74.9
Kr 08-84	1R + 2R	1.45			
Kr 08-156	1BS/1RL	1.45			
Kr 08-52	1R/1D	1.45			
Kr08-20	IR+6R	1.44			
Dragon		1.22			

For TOTE the 20 accessions with highest value and their corresponding alien segments are shown while for %UPP, those higher than the standard (Dragon = Swedish winter wheat).

## Discussion

New sources of genetic diversity are essential to improve yield, root growth, stand establishment, adaptation to climate change, nitrogen use efficiency, water use efficiency, resistance to abiotic and biotic stresses, biomass, photosynthetic potential, nutritional and end-use quality. In this paper, results from studies over a range of years are compiled to highlight the importance of wheat-alien introgression lines as a potential source of several important traits for wheat improvement. Our studies proved that these wheat-alien introgression lines carry various genetic variation e.g. resistance to diseases (rusts, powdery mildew, *S. triticae*, and Fusarium head blight), pests (Hessian fly and aphids), agronomic performance, weed competition, yield potential, microelements (Fe, Zn, Cd, etc.), fertility, alpha amylase activity, and positive end-use quality.

The evaluated 2R (2B) and 2R (2D) substitution lines showed resistance to all stem rust races at both the seedling and adult plant stages. Additionally, three of the 3R (3D) (SLU214, SLU219, and SLU222) substitution lines and seven of the wheat-*T. junceiforme* were found as potential sources of stem rust resistance genes. From the screening of a collection of wheat-alien introgression lines, the line SLU238 [2R (2D) wheat-rye disomic substitution] possessed resistance to many races of *Pgt*, including the widely virulent race TTKSK (Rahmatov et al., 2016a). In previous studies, Rahmatov et al. (2016b), reported that by the crossing of SLU238 and CS *ph1b* mutant, a new wheat-rye Robertsonian translocation 2DS·2RL was developed as the source of the gene *Sr59*. To this date, no stem rust resistance genes have been reported from the 2R chromosome, but chromosome 2R from different rye sources has been described as a source of resistance to various diseases and insects and also various agronomic traits. Previously, the resistance genes to leaf rust *Lr25* and *Lr45*, powdery mildew *Pm7* and Hessian fly resistance gene *H21* have been reported from the 2R chromosome (Friebe et al., 1996; Friebe et al., 1999). Furthermore, the resistance genes *Sr27*, *Sr31*/*Yr9*/*Lr26*, *Sr50*, *Sr1RS* ^Amigo^ and, *SrSatu* have been described, originating from the rye chromosomes 1R and 3R, and these have been found to be effective against many of all the three rusts races (Marais and Marais, 1994; Mago et al., 2002; Singh et al., 2011; Olivera et al., 2013). Out of these resistance genes, *Sr31* has been deployed widely and provided durable resistance against stem rust races for over 30 years in agriculture (Singh et al., 2008).

Agronomic performances of some of the alien-wheat introgression lines were similar to wheat for grain yield, straw length, lodging, grain volume weight, 1000-kernel weight, fertility, grain a-amylase activity, and end-use quality (Hysing et al., 2007; Andersson et al., 2016) while some of the lines showed large variation in agronomic performance. Field studies indicated a correlation between the presence of rye (1R, 2R, 3R, 5R, 1R + 6R) and *L. racemosus* chromosomes, with low level of powdery mildew, *S. triticae* and Fusarium head blight infections (Andersson et al., 2016). Previous studies have reported an *Fhb3* resistance gene to Fusarium head blight derived from *L*. *racemosus* (Qi et al., 2008), which might also be present in our wheat—*L*. *racemosus* introgression lines. Therefore, future evaluation of these lines to other powdery mildew and *Z. triticae* isolates at seedling and adult plant stages are needed. Hysing et al. (2007), reported that red coleoptile color was correlated to the presence of the 2BS.2RL translocation allowing this character to be used as a morphological marker. Furthermore, lines with the 2BS.2RL translocation were demonstrated a high level of resistance against leaf rust and powdery mildew at the seedling stage (Merker and Forsström, 2000; Hysing et al., 2007) and adult plant resistance to TTKSK (Ug99; Rahmatov et al., 2015), thus indicating presence of uncharacterized resistance gene/s. Valuable rye chromosomes harboring beneficial genes from 4R, 5R, and 6R have also been identified (Rahmatov et al., 2017). These lines containing 4R, 5R, and 6R chromosomes are pointed out here as useful due to the fact that they are possessing novel stripe rust resistance genes. Further investigations are needed to understand the underlying genetic basis of this resistance. In various studies, stripe rust and powdery mildew resistance genes have reported on the 4R, 5R, and 6R chromosomes (An et al., 2015; Schneider et al., 2016; Xi et al., 2019), in which *Yr83* was mapped on the 6RL (Li et al., 2020). Besides this, chromosomes 4R and 6R have been demonstrated to contribute increased protein content and also to be associated with good pollinator traits (Nguyen et al., 2015; Schneider et al., 2016). Thus, there is a need to further exploit these wheat-alien introgression lines with various chromosome constitutions for wheat improvement.

High levels of resistance were identified in lines with the 1R, 3RS, 1R + 6R, 5R, and *L. mollis* chromosome introgressions against RWA. Resistances to RWA obtained from the wheat-alien introgression lines particularly lines with the 3R, 5R and *L. mollis* chromosomes have not previously been reported (Andersson et al., 2015). Previously, *Dn7*, *Gb2*, and *Gb6* resistance genes to cereal aphids have been reported on chromosome arm 1R (Friebe et al., 1996; Friebe et al., 1999; Anderson et al., 2003). Also, 1RSam.1AL and MA1S.1RLe(1B), 1Re(1D) wheat–rye translocation, and substitution lines were shown with a high level of resistance against HF and RWA, and these lines are now used in the international wheat breeding programs (Crespo-Herrera et al., 2019). The wheat-alien introgression lines with the presence of 1R, 1RS, 2R, 3R, 3RS, 4R, 5R, 6RL, and *L. racemosus* and *L. mollis* chromosomes provides resistance to the Syrian HF biotype. Previous studies have verified alien germplasm to contribute HF resistance in wheat through the *H21* and *H25* resistance genes from rye, located on 2R and 6R, respectively (Friebe et al., 1999). Hysing et al. (2007), reported that lines with the T2BS.2RL were susceptible to the HF biotypes thus this indicates different rye sources used for developing Swedish wheat-alien introgression lines. Host resistance to these insects is the most effective way of control, and various resistance genes have been derived from alien species. The resistances to RWA and HF reported here originating from alien material have not previously been described and can, therefore, be useful to widen the pool of resistance genes in wheat breeding for resistance to RWA and HF.

The wheat-rye introgression lines displayed a good source of allelopathic potential, while lines with *L. mollis* chromosome showed a low level of allelopathic potential and the bread wheat genotypes showed no allelopathic activity. These wheat-alien lines can be used as a source of allelopathic potential and weed competitiveness in breeding programs to improve weed suppression ability for wheat improvement. Bertholdsson et al. (2012a, b), showed that the highest allelopathic potential was found in lines with 1R and 2R chromosomes. Moreover, some lines with multiple rye chromosomes (1R + 6R and 1R + 4R + 6R + 7R) were also showed high allelopathic activity (Bertholdsson et al., 2012). Previous studies have identified lines with 1R substitution showing early vigour, which can be positive for the root exudation of allelochemicals (Ehdaie et al., 2003). Breeding efforts for the allelopathic potential is considered as a complex trait (Bertholdsson, 2007), although successful examples are present on rice (Kong et al., 2006) and spring wheat (Bertholdsson, 2010). Quantitative trait loci (QTLs) linked to allelopathic traits have found on wheat chromosomes (Wu et al., 2003), thus, this indicates that allelopathic traits inherited quantitatively. The lines with high allelopathic potential identified in this study may be worthwhile for the breeding of allelopathic wheat, particularly for the purpose of organic wheat.

Various zinc, iron, and cadmium concentrations were identified in these lines. Wild relatives of wheat represent a reach source of micronutrient benefits because they have a huge and deep rooting system during its vegetation period that most efficiently uptake micronutrient if they are available in the soil (Borill et al., 2014). This has been proved by using natural genetic diversity for micronutrient uptake that can increase the nutrient content in wheat through genetic improvement (Velu et al., 2014). For instance, studies have indicated high levels of Fe and Zn to be encoded by a *Gpc-B1* locus, present in particular in wild emmer wheat (Uauy et al., 2006; Johansson et al., 2020). Thereby, genetic biofortification in wheat can be enhanced using these wheat-alien introgression lines as a source of natural genetic diversity.

Plant breeding is mostly targeting traits that improve yield potential, i.e. resistance to biotic and abiotic stresses, although for wheat improved baking and bread-making quality is also of outmost importance (Helguera et al., 2020). Wheat flour has, in particularly due to its unique protein properties, qualities which makes it outstanding for end-uses for daily food products such as bread, pastries, biscuits, porridge, cookies, etc. (Johansson et al., 2013). The gluten proteins, the gliadins, and the glutenins, encoded on group 1 and group 6 of the wheat chromosomes, are to a high extent responsible for the impact on the baking quality of wheat (Johansson et al., 2013). Alien introgressions into the wheat genome have often resulted in negative effects on the baking quality, e.g. the *Sec-1*, *Sec-2*, and *Sec-3* genes from rye instead of corresponding wheat genes at the group 1 chromosome of wheat (Kim et al., 2005). However, introgressions of rye from other parts of the genome than from the group 1 chromosomes might have less tremendous effects on the baking quality. Thus, previous results have indicated that 2BS.2RL wheat–rye translocations only had minor effects on baking quality (Hysing et al., 2007). These authors indicated that there were not any significant differences between the translocation and non-translocation groups like for grain a-amylase activity, grain starch, protein content, and other agronomic performances. Bread-making quality is known to be determined to a large extent by the gluten proteins, their amount and distribution (Johansson et al., 2013). Thus, the grain proteins concentration, the specific protein composition, the amount of specific proteins, and the amount and size distribution of polymeric protein are all factors of relevance for the bread-making quality (Finney and Barmore, 1948; Johansson et al., 2002; Johansson et al., 2003; Johansson et al., 2005; Johansson et al., 2008; Johansson et al., 2013). The evaluated alien introgression lines showed a high level of variability in both grain protein concentration and gluten strength. Thus, the alien material evaluated here, seems to have also interesting properties when it comes to specific quality breeding. Introgressions of *Leymus* seem to be able to contribute both high nutrition and high gluten strength to the material.

### Alien Breeding Through Novel Tools

Introgression of desired genes from wild relatives into the bread wheat has become widely recognized as diversifying genetic diversity. However, wheat-alien chromosome additions often contribute negatively to the agricultural value of the line, therefore, desired gene/s has to be transferred into the wheat genome. Such transfers are normally blocked by the presence of a *Ph1* (*Pairing homoeologous*) allele, which strictly controls homologous chromosome pairing across the hexaploid genome to prevent hybridization between wheat and an alien species (Riley and Chapman, 1958). Anyhow, alien chromosome segments carrying gene/s of interest have been widely transferred into the wheat genome using the CS *ph1b* homoeologous recombination, radiation, and embryo culture techniques (Sears, 1977; Sears, 1993; Chen et al., 1994; Merker and Lantai, 1997). These approaches in a combination of molecular and cytogenetic manipulations were used to facilitate the introgression of *Sr26* and *Lr19* from *Thinopyrum ponticum*, *Sr39* from *Aegilops speltoides*, *Sr59* from *S. cereale*, etc. with small alien chromosome segments (Sharma and Knott, 1966; Merker and Lantai, 1997; Niu et al., 2011; Rahmatov et al., 2016a; Rahmatov et al., 2016b). More recently, reference genomes have been made available for wheat, (IWGSC, 2014), rye (Bauer et al., 2017), barley (IBGSC, 2012), rice (IRGSP, 2005), and Brachypodium (IBI, 2010), greatly facilitating the forward and reverse genetics in crops. Various high-throughput genotyping platforms such as the 9K and 90K Illumina Infinium SNP arrays and the 35K and 820K Affymetrix Axiom arrays have been developed for gene and QTL mapping (Wang et al., 2014; Winfield et al., 2016; Allen et al., 2017). In addition, genotyping-by-sequencing and exome capture sequencing opens up more opportunities for markers development and gene isolation (Poland et al., 2012; Krasileva et al., 2017). All these genotyping platforms provide tremendous tools to assess the genetic diversity and allelic variation across plant genomes. However, a low level of SNP polymorphism between hexaploid wheat and wild relatives has been reported which negatively impact the use of the mentioned platforms (Winfield et al., 2016). Therefore, Tiwari et al. (2014) suggested the use of flow cytometric chromosome sorting to develop unique SNP markers for the mapping of alien genes to overcome these challenges. Whole-genome shotgun sequencing is becoming another valuable breeding tool in terms of time and cost, which are already used in major crops such as wheat (Brenchley et al., 2012), maize (Hufford et al., 2012), rice (Huang et al., 2012), and soybean (Fang et al., 2017). However, the transfer of desired alien gene/s remains a challenge, although some advances have been made in transferring resistance genes. Jupe et al. (2013) developed an exome capture and sequencing of nucleotide-binding leucine-rich repeat (NLR) genes in potato. Such resistance gene enrichment sequencing (RenSeq) allowed a rapid cloning of the *Sr22* and *Sr45* resistance genes through mutational genomics (Steuernagel et al., 2016). Another approach, MutChromSeq, has been applied through mutational genomics, chromosome flow sorting and sequencing that has resulted in the cloning of the *Pm2* resistance gene (Sánchez-Martín et al., 2016). Interestingly, another cloning approach suggested a combination of association genetics and R gene enrichment sequencing, which rapidly identified stem rust resistance genes for cloning (Arora et al., 2019). Besides, a combination of cisgenesis and genome editing tools may accelerate the plant breeding process (Cardi, 2016). Also, the use of speed breeding may significantly accelerate the generation times and breeding cycles (Watson et al., 2018). Therefore, integration of high-throughput genotyping and precise phenotyping tools may efficiently assist in transferring the introgression of small alien chromatin segments to develop new genetic diversity for wheat improvement. For example, the development of synthetic wheat and 1RS chromosome arm has made a great contribution to sustainable wheat production. Evidently, for the development of superior crop cultivars requires new genetic variation that meets sustainable agriculture and food security needs.

## Conclusions—Alien Genes Into Modern Wheat—Future Perspectives

Every day, the human population is growing, and with that the demand of food from sustainable and healthy crop production. To adequately meet the global food demand required by 2050, there is a need to increase wheat yield annually. These can be achieved through the two unique opportunities; plant breeding and improved agronomic practices. Importantly, to meet projected food demand, the breeding programs need to broaden the existing genetic base, in particular by the use of alien species with the potential to improve yield, resistance to biotic and abiotic stresses and quality. Several of our studies have identified new sources of resistance to fungal diseases and insects in the wheat-alien introgression derivatives from *S. cereale*, *L. mollis*, *L. racemosus* and *T. junceiforme*. Also, these lines exhibiting good agronomic performances, high allelopathic potential, and superior end-use quality traits. Our results suggest that some of the lines could be used as a source of high Iron and Zinc and low Cadmium concentrations. These findings show that the wheat-alien introgressions are a potentially useful genetic resource for wheat improvement. The introgression of large alien chromosomes usually challenges researchers and breeders by causing linkage drag that can negatively effect on yield and quality properties. Fortunately, with the presence of high-throughput genotyping and phenotyping tools, opportunities increase to transfer desired gene/s with a small alien chromosome segment. Consequently, research is currently underway to transfer stem and stripe rust resistance genes into the elite wheat background to be used by breeders to develop superior wheat cultivars with new resistance genes. Further, additional research is also in progress for characterization and transferring of useful traits such as micronutrients (Zn, Fe, and Cd), allelopathic potential, diseases, and insect resistance as well as stable baking quality.

## Data Availability Statement

The datasets presented in this study can be found in online repositories. The names of the repository/repositories and accession number(s) can be found in the article/supplementary material.

## Author Contributions

EJ, TH, SA and MR planned various parts of the study, the hypothesis, and the objectives. TH, MP-L, SA, RA carried out various parts of the field and lab work. All authors contributed to compiling various parts of the results. EJ and MR planned the writing of this paper and did the first draft. All authors contributed to the article and approved the submitted version.

## Funding

The study was funded by Partnership Alnarp grant nos. PA710 and PA1094, the Swedish Research Council VR grant no. 2016-05806 and Formas grant no. 2017-00514.

## Conflict of Interest

The authors declare that the research was conducted in the absence of any commercial or financial relationships that could be construed as a potential conflict of interest.
